# Efficacy of methotrexate and etanercept biosimilar rhTNFR:Fc in Chinese patients with active rheumatoid arthritis: A controlled, randomized and multicenter study

**DOI:** 10.1038/s41598-020-64991-5

**Published:** 2020-08-31

**Authors:** Qingjun Wu, Yan Zhao, Dong Xu, Zhuoli Zhang, Zhenbin Li

**Affiliations:** 1 Department of Rheumatology, Peking Union Medical College Hospital, Beijing, China; 2 Department of Rheumatology, Peking University First Hospital, Beijing, China; 3 Department of Rheumatology, Bethune International Peace Hospital, Shijiazhuang, Hebei Province China

**Keywords:** Randomized controlled trials, Rheumatoid arthritis

## Abstract

Rheumatoid arthritis is a chronic inflammatory disease which could lead to severe joint damage and disability. This study was performed to determine the efficacy and safety of methotrexate (MTX) therapy combined with maintenance or discontinuation of etanercept biosimilar rhTNFR:Fc in active rheumatoid arthritis patients in Chinese patients. In this controlled, randomized and open-label study, 89 patients with active rheumatoid arthritis were enrolled at 7 institutions in China between September 2010 and May 2011. In a period of 52 weeks, patients were randomly assigned to one of three treatment groups: MTX plus rhTNFR:Fc for 52 weeks, MTX plus rhTNFR:Fc for 24 weeks, or MTX monotherapy. The primary endpoint was the joint damage evaluated by change from baseline (CFB) of van de Heijde modified Total Sharp Score (mTSS). Intention-to-treat population were used for analysis. A total of 89 enrolled patients were eligible for this study, of whom 32 were assigned to MTX plus rhTNFR:Fc52 group, 31 to MTX plus rhTNFR:Fc24, and 26 to MTX monotherapy. Only one patient was lost to follow up in the MTX plus rhTNFR:Fc24 group. The mTSS CFB was lower in the rhTNFR:Fc pooled group (combination of data in the MTX plus rhTNFR:Fc52 group and MTX plus rhTNFR:Fc24 group) comparing with MTX monotherapy at week 24 and 52 (P = 0.03 and P < 0.01). Additionally, ACR50 and ACR70 response rates were both higher in the rhTNFR:Fc pooled group than MTX monotherapy (P < 0.05). Combination of MTX and rhTNFR:Fc in patients with active rheumatoid arthritis could effectively inhibit joint structure damage.

## Introduction

Rheumatoid arthritis, a chronic inflammatory disease, could lead to severe joint damage and disability. The prevalence of rheumatoid arthritis is 5 in every 1,000 adults worldwide with women being 2–3 times more likely than men to develop rheumatoid arthritis regardless of age^1,2^. Treatment goal of rheumatoid arthritis was to achieve clinical remission and to prevent joint damage^3^.

Early therapy with disease-modifying anti-rheumatic drugs (DMARDs) could improve the outcome of rheumatoid arthritis. Methotrexate (MTX) is one of the most important conventional DMARDs as first-line therapy, which has been used for about 50 years in patients with rheumatoid arthritis. The potential mechanisms of action of methotrexate for rheumatoid arthritis include antagonism of folate-dependent processes, generation of reactive oxygen species, stimulation of adenosine signaling, inhibition of methyl-donor production, downregulation of adhesion-molecule expression, eicosanoids and matrix metalloproteinases and modification of cytokine profiles^4^. Targeted therapies such as tumor necrosis factor inhibitor (TNFi) and Janus kinase inhibitors were recommended when first-line therapy such as MTX fails^1,5,6^. TNFi include etanercept, adalimumab, certolizumab, golimumab and infliximab. Etanercept is a recombinant human TNF receptor (p75) Fc fusion protein, which has been widely used in clinical practice^7^. Combination of etanercept and MTX have been found to improve radiographic and clinical outcomes compared to MTX monotherapy for rheumatoid arthritis^8,9^. In a 24-week, double-blind trial, more rheumatoid patients receiving combination of etanercept and methotrexate met American college of rheumatology (ACR) 20 and 50 criteria comparing with MTX monotherapy^10^. Data from DREAM registry showed that TNFi in combination with MTX had better disease activity score of 28 joints (DAS28) and health assessment questionnaire (HAQ) values than TNFi monotherapy^11^. In COMET trail, one year of combined treatment of etanercept and MTX could reach clinical remission and radiographic non-progression in early severe rheumatoid arthritis^12^.

Recombinant human TNF-α receptor II:lgG Fc fusion protein (rhTNFR:Fc, YISAIPU^®^, 3SBio Inc., China) is an etanercept biosimilar as TNFi for moderated and serious active rheumatoid arthritis. The rhTNFR:Fc has been widely used in clinical practice in China for 14 years. Beside rheumatoid arthritis, active ankylosing spondylitis was also the indication of rhTNFR:Fc^13,14^. Comparing with MTX, rhTNFR:Fc revealed more disease activity improvement in Chinese rheumatoid arthritis patients^15^.

In the view of societal perspective, long-term medication of TNFi was not reality and discontinuing of TNFi was considered in the clinical practice. But when to discontinue TNFi in the treatment was still unknown. In this study, we report the results of a randomized, controlled, open-label and multicenter trial to address the effectiveness and safety of MTX in combination with rhTNFR:Fc for 52 weeks or 24 weeks on the active rheumatoid arthritis in Chinese patients.

## Methods and Patients

### Study design

**Figure 1:**
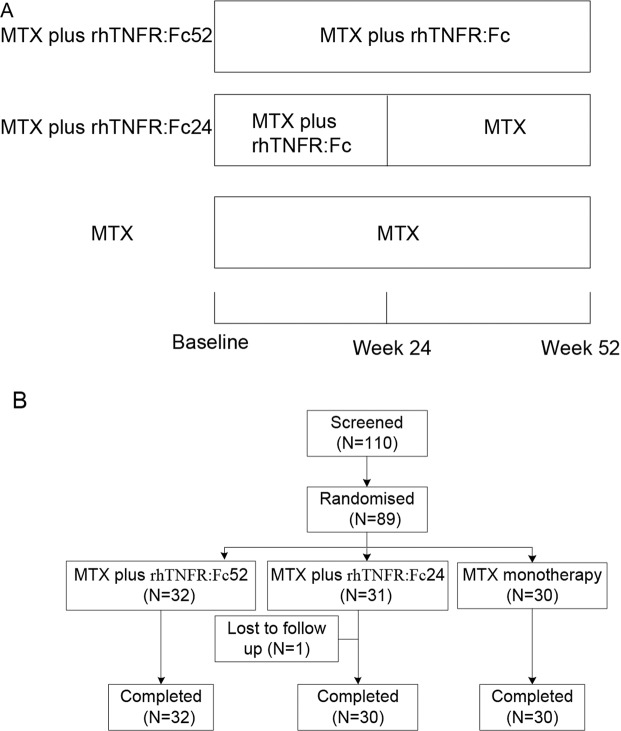
(**A**) Schematic description for effeicacy and safety of MTX plus 24-week and 52-week rhTNFR:Fc versus MTX alone in patients with active rheumatoid arthritis. (**B**) Patient disposition. rhTNFR:Fc, recombinant human tumor necrosis factor-α receptor II:lgG Fc fusion protein injection; MTX, methotrexate.

A controlled, randomized, open-label and multicenter study was carried out at 7 institutions (including Peking Union Medical College Hospital, Peking University First Hospital, Bethune International Peace Hospital, Nanjing Drum Tower Hospital, The Second Affiliated Hospital of Sun Yat-Sen University, West China Second University Hospital, Sino-Japanese Friendship Hospital of Jilin University) in China from October 30, 2010 to January 30, 2013 (chictr.org.cn identifier number is ChiCTR1900024107 and the date of registration were on June 26^th^, 2019; information about registration are available at chictr.org.cn). Active rheumatoid arthritis patients were randomly assigned to one of three treatments for up to 52 weeks at a 1:1:1 ratio using centre-stratified block-permuted randomization: MTX (10 mg per week at initial and increased to 15 mg at the fourth week) plus rhTNFR:Fc (25 mg subcutaneous injections twice weekly) for 52 weeks; MTX (same with that in MTX plus rhTNFR:Fc52 group) plus rhTNFR:Fc (same with that in MTX plus rhTNFR:Fc52 group) only for the first 24 weeks and then MTX continued till week 52; MTX monotherapy (Fig. 1A). Addition of folic acid (5 mg per week) was introduced after MTX administration.

This study was approved by the ethics review board of Peking Union Medical College Hospital (NO. S328). Besides, ethics review boards in the other 6 institutions also approved the protocol of this study. Protocol was conducted in accordance with Declaration of Helsinki and Good Clinical Practice Guidelines. All participating patients signed written informed consent before being recruited.

### Patients

Eligible patients, aged 18 to 60 years old, were included if they met the following criteria: rheumatoid arthritis diagnosed according to 1987 ACR classification criteria for rheumatoid arthritis for at least 6 months; ≥8 tender joints (of 68 joints assessed); ≥6 swollen joints (of 66 joints assessed); erythrocyte sedimentation rate (ESR) ≥ 28 mm/h; C-Reactive Protein (CRP) ≥ 20 mg/L; ≥1 X ray erosion on hands or wrist at screening and baseline; stable doses of oral corticosteroids (≤10 mg/day of prednisone or equivalent) and non-steroidal anti-inflammatory drug (up to the maximum recommended dose) for ≥ 4 weeks prior to baseline.

Exclusion criteria included prior biologic treatments; surgery within 8 weeks before initiation of study medication or within 6 months after initiation; stage IV rheumatoid arthritis by wrist X-ray; any vaccination within 4 weeks; other rheumatic autoimmune disease such as systemic lupus erythematosus; history of malignancy; other inflammatory arthritis such as psoriatic arthritis; active or recurrent of infection; history of serious/chronic infection treated by antibiotics within 4 months; nervous system diseases; serious and uncontrolled cardiovascular diseases, pulmonary diseases, renal diseases, liver diseases, endocrine and gastrointestinal diseases; and pregnant or breast-feeding women.

### Study endpoints

The patientsdpointsngastrointestinal diseasesdiseaseantiline, week 12, week 24 and week 52. The primary efficacy end point was designated as the change from baseline (CFB) in van de Heijde modified Total Sharp Score (mTSS) at week 24 and 52. The mTSS CFB was based on the joint space narrowing and joint erosion evaluated independently by well-trained X-ray readers who were blinded to the treatment of each patient. Secondary endpoints included remission rates (disease activity score, DAS28 < 2.6; clinical disease activity index, CDAI ≤ 2.8; simplified disease activity index, SDAI ≤ 3.3) at week 12, 24 and 52 and ACR response rates (ACR 20, ACR 50 and ACR 70). Safety endpoints included all adverse events and serious adverse events.

### Statistical analysis

A sample size of 90 patients was estimated with the assumption that mean mTSS CFB was −0.5 ± 1.0 in the MTX plus rhTNFR:Fc group, and 2.5 ± 5.0 in the MTX group at week 52 based on previous reports^12,16^. A sample size of 25 patients per treatment group was calculated to be necessary for more than 80% power with an combined, Assuming drop-out of patients, the study required a minimum of 30 patients in each group.

Descriptive statistics were calculated for baseline clinical and demographic characteristics. Three groups were compared using analysis of variance (ANOVA) or Kruskal-Wallis tests for continuous variables. ACR response and remission rates were assessed by Cochran-Mantel-Haenszel tests. Pairwise comparison was performed using LSM tests or Nemenyi test for continuous variables such as mTSS CFB or Pearson’s arson mTSSntinuou’s exact test for categorical variables such as ACR and remission rates. Two groups were compared using two-sample t tests or Wilcoxon rank-sum test for continuous variables or Pearson’s o groups were com’s exact test for categorical variables. Intention-to-treat (ITT) analyses were undertaken on the full analysis set (FAS), which included all eligible patients who received at least one study medication and had at least one post-therapy assessment of efficacy. The last observation carried forward (LOCF) method was used to impute any missing values. The safety analysis included all patients who received at least one study medication and had at least one post-therapy assessment of efficacy. All analyses were performed on the SAS 9.2 software.

## Results

### Patient disposition and demographics

A cohort of 110 patients from 7 centers were screened and 89 were randomized in a 52-week period to MTX plus rhTNFR:Fc for 52 weeks (N = 32), MTX plus rhTNFR:Fc for 24 weeks (N = 31) or MTX monotherapy (N = 26). Of these, all patients completed the 52-week period and only one patient was lost to follow up in MTX plus rhTNFR:Fc24 group (Fig. 1A). Demographic and clinical characteristics were generally well balanced among these three groups except rest pain visual analogue score (VAS) and HAQ (Table 1). Clinical characteristics were generally well balanced between rhTNFR:Fc pooled groups (data in MTX plus rhTNFR:Fc52 group and MTX plus rhTNFR:Fc24 group were pooled) and MTX group except rest pain VAS, HAQ, CRP and CDAI (Table 1).

**Table 1:** Demographics and disease characteristics of patients with rheumatoid arthritis at baseline.

	MTX plus rhTNFR:Fc52(n = 32)	MTX plus rhTNFR:Fc24(n = 30)	MTX (n = 26)	P^a^	MTX plus rhTNFR:Fc(n = 62)	P^b^
Female, n (%)	28 (87.50)	22 (73.33)	22 (84.62)	0.32	50 (80.65)	
Age, years	40.78 (13.35)	44.67 (11.73)	43.31 (9.93)	0.43	42.66 (12.64)	
Duration of RA, years	10.73 (9.92)	7.63 (9.14)	7.10 (7.66)	0.22	9.23 (9.60)	
Duration of morning stiffness, min	91.29 (92.47)	111.33 (87.05)	101.15 (136.55)	0.38	101.15 (89.67)	0.36
Rest pain VAS	62.66 (23.83)	63.83 (16.38)	49.27 (25.32)	0.03	63.23 (20.41)	0.02
HAQ	1.62 (0.76)	1.52 (0.56)	1.20 (0.59)	0.04	1.57 (0.66)	0.01
Physician VAS	64.19 (15.66)	65.97 (14.34)	57.69 (17.68)	0.18	65.07 (14.93)	0.07
Patient VAS	64.41 (16.92)	67.63 (16.53)	60.38 (18.54)	0.30	65.97 (16.67)	0.24
Tender joint count (68)	19.06 (13.30)	17.67 (10.77)	13.96 (7.15)	0.21	18.39 (12.07)	0.08
Swollen joint count (66)	11.59 (9.10)	11.60 (6.56)	10.92 (6.74)	0.60	11.60 (7.91)	0.41
ESR, mm/h	51.59 (21.94)	56.13 (29.20)	63.00 (35.17)	0.61	53.79 (25.60)	0.37
CRP, mg/L	3.16 (2.81)	3.97 (3.17)	6.12 (4.15)	0.20	3.55 (2.99)	<0.01
mTSS	14.76 (19.65)	10.17 (13.37)	13.38 (11.00)	0.36	12.68 (17.10)	0.30
Joint erosion	6.86 (9.37)	5.08 (7.40)	6.52 (5.90)	0.27	6.06 (8.50)	0.27
Joint space narrowing	7.90 (10.86)	5.08 (6.59)	6.86 (6.38)	0.64	6.62 (9.20)	0.44
DAS28	4.94 (0.82)	5.15 (0.82)	4.88 (0.91)	0.44	5.05 (0.82)	0.40
CDAI	33.10 (11.61)	35.56 (11.52)	29.65 (11.45)	0.08	34.29 (11.53)	0.04
SDAI	36.26 (12.49)	39.40 (12.56)	35.77 (13.21)	0.35	37.78 (12.52)	0.57

All values are mean (SD) unless otherwise stated. RA, rheumatoid arthritis; VAS, visual analogue score; HAQ, health assessment questionnaire; ESR, erythrocyte sedimentation rate; CRP, C-reactive protein; mTSS, van de Heijde modified Total Sharp Score; DAS28, disease activity score 28; CDAI, clinical disease activity index; SDAI, simplified disease activity index. Pa: comparison among three groups; Pb: comparison between rhTNFR:Fc pooled groups and methotrexate group.

### Primary endpoint

**Figure 2:**
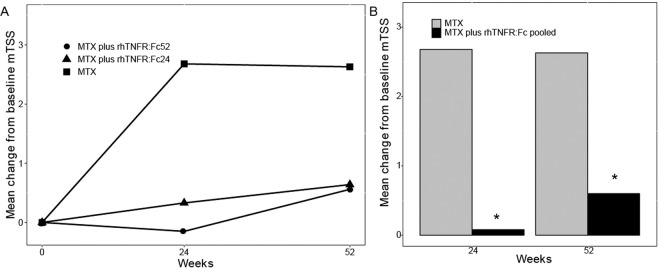
(**A**) The mTSS CFB at week 24 and 52 in MTX plus rhTNFR:Fc52 group, MTX plus rhTNFR:Fc24 group and MTX group. Values in the figure indicate mean at each time point. (**B**) The mTSS CFB at week 24 and 52 in MTX plus rhTNFR:Fc group (data in the MTX plus rhTNFR:Fc52 group and MTX plus rhTNFR:Fc24 group were pooled) and MTX monotheray group. All data were analysed using FAS and LOCF data set. rhTNFR:Fc, recombinant human tumor necrosis factor-α receptor II:lgG Fc fusion protein injection; MTX, methotrexate; mTSS CFB, change from baseline of van de Heijde modified Total Sharp Score (mTSS); FAS, full analysis set; LOCF, last observation carried forward. *P < 0.05.

The mTSS CFB (mean ± SD) at week 52 was 0.56 with MTX plus rhTNFR:Fc52 group, 0.64 with MTX plus rhTNFR:Fc24 group and 2.63 with MTX group. There were significant differences between three groups in terms of mTSS CFB at week 52 (P < 0.01, Fig. 2A). The mTSS CFB in the rhTNFR:Fc24 or rhTNFR:Fc52 group was not significantly lower than that in the MTX monotherapy. Besides, there were no significant differences between MTX plus rhTNFR:Fc52 group and MTX plus rhTNFR:Fc24 group regarding mTSS CFB over the course of treatment (week 24 and 52). Thus, we pooled the data of the two rhTNFR:Fc groups to compare with MTX monotherapy group, which showed that mTSS CFB in the rhTNFR:Fc pooled group was significantly lower than MTX monotherapy group at week 24 and 52 (P = 0.03 and P < 0.01, Fig. 2B).

### Secondary endpoints

**Figure 3:**
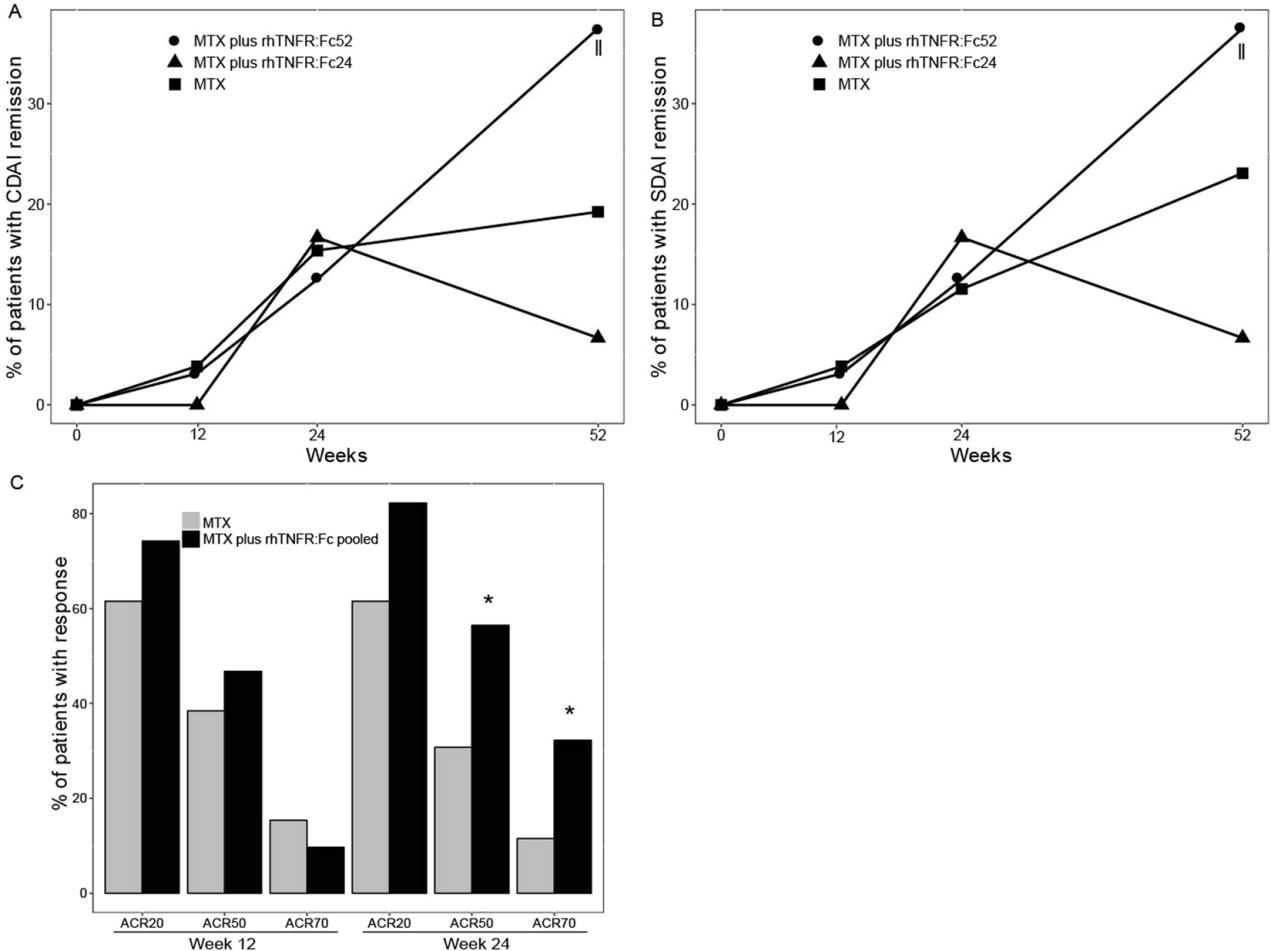
(**A**,**B**) Clinical remission rates at weeks 12, 24 and 52 by CDAI and SDAI response in MTX plus rhTNFR:Fc52, MTX plus rhTNFR:Fc24 and MTX montherapy group. (**C**) Time course of response rates in ACR20, ACR50, ACR70 at week 12 and 24 in MTX plus rhTNFR:Fc pooled group (data in the MTX plus rhTNFR:Fc52 group and MTX plus rhTNFR:Fc24 group were pooled) and MTX group. All data were analysed using FAS and LOCF data set. rhTNFR:Fc, recombinant human tumor necrosis factor-α receptor II:lgG Fc fusion protein injection; MTX, methotrexate; ACR, American College of Rheumatology; CDAI, clinical disease activity index; SDAI, simplified disease activity index; FAS, full analysis set; LOCF, last observation carried forward. ǁMTX plus rhTNFR:Fc52 versus MTX plus rhTNFR:Fc24 group, P < 0.01; *MTX plus rhTNFR:Fc pooled group versus MTX group, P < 0.05.

Proportion of rheumatoid arthritis patients with CDAI and SDAI remission at week 52 was significantly higher in the rhTNFR:Fc52 group comparing with rhTNFR:Fc24 group (P < 0.01 and P < 0.01, Figs. 3A and 3B). DAS28 remission rate in the rhTNFR:Fc52 group or rhTNFR:Fc24 group was not significantly higher than MTX monotherapy (data not shown). As ACR response rates (ACR20, ACR50 and ACR70) were not significantly higher in the rhTNFR:Fc52 group than that in the rhTNFR:Fc24 group (data not shown) at week 12 and 24, data in the two rhTNFR:Fc groups were pooled for further analysis. ACR50 response rate at week 24 in the rhTNFR:Fc pooled group and MTX group were 56.45% and 30.77% (P < 0.05), respectively and ACR70 response rates were 32.26% and 11.54% (P < 0.05), respectively (Fig. 3C).

### Safety analysis

The occurrence of adverse events was similar across all three treatment groups (Table 2). A total of five patients had adverse events in MTX plus rhTNFR:Fc52 group: two patients had urinary tract infection; one had tuberculosis (to be confirmed), one had nausea and one had diarrhea. Four patients had adverse events in the MTX plus rhTNFR:Fc24 group: one had herpes zoster; one had cough with yellow sputum; two had abnormal liver function. A total of four adverse events were reported in MTX group, of which there was one with palpitation, two with abnormal liver function and one with anemia.

**Table 2:** Safety summary.

	MTX plus rhTNFR:Fc 52 group(N = 32)	MTX plus rhTNFR:Fc 24 group (N = 30)	MTX group(N = 26)
Herpes zoster	0	1	0
Urinary tract infection	2	0	0
Cough with yellow sputum	0	1	0
Tuberculosis	1	0	0
Palpitation	0	0	1
Nausea	1	0	0
Diarrhea	1	0	0
Abnormal liver function	0	2	2
Anemia	0	0	1

## Discussion

In this controlled, randomized and open-label study, we observed that combination of MTX and rhTNFR:Fc significantly inhibited joint damage (as judged with mTSS CFB) comparing with MTX monotherapy in Chinese patients with active rheumatoid arthritis. This finding was similar with previous studies^8,12^, indicating that combination of etanercept and MTX therapy was superior to MTX monotherapy with radiographic non-progression for patients with early severe active rheumatoid arthritis.

In rheumatoid arthritis, accumulation of joint damage induced by insufficiently treatment could lead to disability^1^. The mTSS score was evaluated based on the joint erosion and space narrowing score by X-ray analysis. In our study, combination of rhTNFR:Fc and MTX could inhibit the joint damage over the treatment course of 52 weeks. TNFi has been considered to be combined with conventional synthetic DMARDs for rheumatoid arthritis by European League Against Rheumatism and Asia-Pacific League of Associations for Rheumatology^5,6^. Many clinical trials in patients with rheumatoid arthritis have shown the superiority of MTX in combination with anti-TNF therapy comparing to MTX monotherapy^17–22^. However, this study was first to evaluate the efficacy of MTX in combination with rhTNFR:Fc on rheumatoid arthritis in Chinese population in terms of joint damage inhibition, providing evidence for the combination of rhTNFR:Fc and MTX therapy in clinical practice. In this study, we also want to investigate if the 24-week treatment is enough for rhTNFR:Fc to take effect. However, there was no significant difference between two rhTNFR:Fc groups in terms of joint damage inhibition. But MTX plus rhTNFR:Fc24 group or MTX plus rhTNFR:Fc52 group has similar efficacy results with MTX monotherapy. Thus, further study is needed to investigate the optimal time of rhTNFR:Fc discontinuation in the treatment of rheumatoid disease.

Additionally, 37.5% of the patients in MTX plus rhTNFR:Fc52 group achieved remission (judged by CDAI or SDAI) at week 52 while only about 20% of patients achieved remission in the MTX group. In the open-label PRESERVE trial, about 23.7% of patients with moderately active rheumatoid arthritis receiving MTX and 50 mg etanercept achieved CDAI remission at week 36^23,24^. For rhTNFR:Fc pooled group, ACR 50 (56.45%) or ACR70 response rate (32.26%) was significantly higher than that in the MTX monotherapy group at week 24. The results were consistent with previous report that the ACR50 response rate (from 27% to 70%) of combined strategy (MTX plus biologics) was superior to oral MTX^25^. These findings suggest that substantial benefit was attained by MTX in combination of rhTNFR:Fc.

The reasons of additive effect of MTX may be that the potential mechanisms of action of MTX in the rheumatoid arthritis are totally different from rhTNFR:Fc^4^. MTX reduced the interleukine-6 but not TNF-ay be that s attained by Koenig, A. S., Jone^26^.

There were no serious adverse events in this study. Due to the limited size of this study, incidence was not compared among these three groups. The safety findings showed that differences among these groups might not be a concern for clinicians to consider MTX in combination with rhTNFR:Fc. Similarly, adverse events were mild in previous study using the rhTNFR:Fc^15^.

In this study, the maximum dose of MTX was 15 mg once week, which was lower than 20 mg or 25 mg once week in the previous studies^10,12,23^. Thus, the dose reduction of MTX in our study could minimize the MTX toxicity. There were several limitations in our study. Firstly, there was not sufficient power to detect differences between the two rhTNFR:Fc groups. MTX plus rhTNFR:Fc52 group has similar result with MTX plus rhTNFR:Fc24 group regarding primary endpoint in our study. Secondly, for limited fund, open-label design was adopted in this study, which may bring assessment bias.

The results of this study suggest that joint damage inhibition is an achievable goal in Chinese patients with active rheumatoid arthritis by combination of MTX and rhTNFR:Fc. Besides, MTX and rhTNFR:Fc combination therapy was well tolerant. However, large-scale prospective cohort study could be performed to investigate the impact of maintenance or discontinuation of rhTNFR:Fc during rheumatoid arthritis treatment on the radiographic progression in the future.

## Supplementary information

Clinical trial protocol.

## Data Availability

All data generated or analyzed during this study are available at www.chictr.org.cn. This study was sponsored by 3SBio Inc, China.
