# Supplementary material for: Efficacy of methotrexate and etanercept biosimilar rhTNFR:Fc in Chinese patients with active rheumatoid arthritis: A controlled, randomized and multicenter study
Source: Sci Rep. 2020 Aug 31;10:14270. doi: 10.1038/s41598-020-64991-5 (PMC7458921; doi:10.1038/s41598-020-64991-5)
Supplement: Supplementary file 1 — Clinical trial protocol. [file 41598_2020_64991_MOESM1_ESM.doc]

**A randomized, controlled, open label, multicentric clinical trial to evaluate the efficacy and safety of recombinant tumor necrosis factor receptor:Fc fusion protein with methotrexate in active rheumatoid arthritis (ReABLE-II)**

**Study Protocol**

| □ 01 | Peking Union Medical College Hospital |
| --- | --- |
| □ 02 | Peking University First Hospital |
| □ 03 | Bethune International Peace Hospital |
| □ 04 | Nanjing Drum Tower Hospital |
| □ 05 | The Second Affiliated Hospital of Sun Yat-sen University |
| □ 06 | West China Hospital of Sichuan University |
| □ 07 | China-Japan Union Hospital of Jilin University |

**April 2010**

**Summary**

**Title:**

A randomized, controlled, open label, multicentric clinical trial to evaluate the efficacy and safety of recombinant tumor necrosis factor receptor:Fc fusion protein (rhTNFR:Fc) with methotrexate (MTX) in active rheumatoid arthritis (ReABLE-II)

**Trial Objective:**

Compare the clinical efficacy and the influence on the joint radiology progression between different treatment groups of RhTNFR:Fc combined with MTX and between these groups and the MTX monotherapy.

**Trial Design:**

A randomized, controlled, open-label, multicenter clinical trial.

**Study Subjects:**

Rheumatoid arthritis subjects who meet the inclusion/exclusion criteria randomly divided into 3 groups (Group A, Group B and Group C) using centre-stratified block-permuted randomization with 30 patients in each group and 90 patients in total.

1. Inclusion criteria: Only those subjects who meet all of the following conditions could be included in the trial
2. The subjects diagnosed with rheumatoid arthritis according to the revised 1987 American College of Rheumatology (ACR) Criteria, and with a disease duration of ≥ 6 months.
3. During the screening period and the baseline period, the swollen joint count (SJC) of the subjects should be ≥ 6 (66 joints counted), while the tender joint count (TJC) should be ≥ 8 (68 joints counted); ESR ≥ 28mm/h or CRP ≥ 20 mg/l in laboratory examination; and ≥ 1 bone erosion lesions revealed in X-ray films of both hands/wrists.
4. Subjects aged between 18 and 60 (inclusive) years old;
5. There has been no use of any marketed or non-marketed biological agents for rheumatoid arthritis (focusing on: etanercept, infliximab, adalimumab, rituximab[CD20 McAb], tocilizumab[IL-6 receptor Ab], abatacept[CTLA4-Ig], anakinra[IL-1 antagonist]) before screening;
6. Any DMARDs drugs used other than MTX have been stopped for 4 weeks before the trial; Oral cortical hormones (prednisone ≤ 10 mg/d or its equivalent) and NSAIDs (up to the maximum recommended dose) are allowed, but the dose should be stable for at least 4 weeks before the baseline period;
7. Women of childbearing potential agreeing to take effective methods of contraception during the trial;
8. The urine pregnancy test of women of childbearing age should be negative during screening.
9. Subjects willing to sign the nformed consent form (ICF) and comply with the requirements of the study protocol;
10. Exclusion criteria: Subjects complying with any of the following conditions should be excluded from the trial.
11. Major surgery (including joint surgery) performed within 8 weeks before screening, or major surgery planned within 6 months after enrollment.
12. Patients with rheumatic autoimmune diseases other than rheumatoid arthritis: including systemic lupus erythematosus (SLE), mixed connective tissue disease (MCTD), scleroderma and polymyositis. However, subjects with Sjogren syndrome are allowed to participate in this trial. If ANA is positive, the possibility of SLE should be excluded based on the clinical conditions and the negative result of the anti-DNA antibody test.
13. Rheumatoid arthritis with wrist X-ray being Stage IV.
14. Patients having suffered or suffering from inflammatory arthritis other than rheumatoid arthritis (such as gout, reactive arthritis, psoriatic arthritis, seronegative spondyloarthropathy, Lyme disease).
15. Patients who have received any investigational drug within 4 weeks before screening (or 5 half-life periods, whichever is the longer).
16. Patients who have undergone any cell elimination therapy, including the investigational drug (e.g. CAMPATH, anti-CD4, anti-CD5, anti-CD3 and anti-CDl9).
17. Patients treated with intravenous gamma globulin, plasma exchange or Prosorba plasma separation exchange within 6 months before the baseline,.
18. Patients who have been injected with cortical hormones intraarticularly or by other means within 4 weeks before the baseline.
19. Patients who have received any vaccine within 4 weeks before the baseline.
20. Patients who have used any alkylating agents, such as cyclophosphamide, chlorambucil or total lymphoid irradiation, within 4 weeks before the baseline.
21. Subjects with severe and uncontrolled cardiovascular diseases, neurological diseases, pulmonary diseases (including obstructive pulmonary disease and interstitial lung disease), nephropathy, liver diseases, endocrine diseases (including uncontrolled diabetes) and gastrointestinal diseases, etc.
22. Patients with uncontrolled disease state, such as asthma, psoriasis, inflammatory bowel disease and other diseases, usually requiring oral administration or injection of cortical hormones to treat recurrence.
23. Patients with known currently active or recurrent bacterial, viral, fungal, mycobacterial diseases or other infectious diseases (including but not limited to tuberculosis and atypical mycobacterial diseases, granulomatous diseases shown by chest X-ray, hepatitis B and C, HIV infection, herpes zoster, but excluding onychomycosis infection), or patients requiring hospitalization and intravenous antibiotic therapy within 4 weeks prior to screening or infected patients requiring oral antibiotic therapy within 2 weeks prior to screening.
24. Subjects with a history of malignant tumors, including solid tumors and hematological malignancies (except for patients with cutaneous basal cell carcinoma that has been excised or cured).
25. Pregnant or lactating (breastfeeding) women.
26. Subjects suffering from neuropathy and other painful diseases that may interfere with pain evaluation.
27. Serum creatinine > 1.5 mg/dl.
28. Alanine aminotransferase (ALT) or aspartate aminotransferase (AST) > 2 x ULN (if ALT or AST is measured to be > 2 x ULN for the first time, sampling should be conducted again during the screening period), or total bilirubin > 1 x ULN (if total bilirubin is measured to be > 1 x ULN for the first time, sampling should be conducted again during the screening period).
29. Platelet count < 100 × 10^9^ /L or WBC < 3 × 10^9^ /L.
30. HBsAg or anti-HCV positive

**Test Method:**

The trial is a randomized, controlled, open-label, multicenter clinical study. Qualified subjects after screening will be randomized into 3 groups (Groups A, B and C) using centre-stratified block-permuted randomization, with 30 patients in each group and 90 patients in total:

Group A:

(RhTNFR:Fc + MTX group), 52 weeks; n=30

Group B:

"MTX montherapy"; n=30

(RhTNFR:Fc + MTX group)

Group C:

"MTX monotherapy"; n=30

Baseline

52 weeks

26 weeks

4 weeks

13 weeks

36 weeks

MTX dose increase stage: MTX will be increased from 10 mg/ week to 15 mg/ week

Note: Folic acid at 5mg/ week will be given on Day 2 of MTX administration in the trial.

**Efficacy Observation Indicators:**

Before treatment, 12, 24, 36 and 52 weeks after treatment, the rest pain, morning stiffness duration, swollen joint count and tender joint count , and health assessment (HAQ), subject's assessment of disease, and doctor's assessment of illness will be evaluated.

CRP and ESR will be evaluated before treatment and at 12, 24, 36 and 52 weeks after treatment.

X-ray changes of both wrists (Sharp score) before treatment and 24 and 52 weeks after treatment.

If necessary, MRI before treatment and at Week 52 (15 patients in Group A and 15 patients in Group C respectively; 30 patients in total).

**Efficacy Evaluation:**

ACR20\50\70, DAS28, CDAI and SDAI will be compared within and between the groups at week 24 and 52 week after treatment, and Sharp score changes will be compared.

**Safety Observation Indicators:**

Blood routine, liver and kidney function, ANA, electrocardiogram, chest radiography, etc.

**Safety Evaluation:**

1. Nature and frequency of adverse reactions.
2. Clinically significant changes in laboratory tests.

**Clinical Study Plan**

**1. Brief description:**

This trial is an open, multi-center clinical trial to evaluate the efficacy and safety of recombinant tumor necrosis factor receptor:Fc fusion protein (rhTNFR:Fc) combined with methotrexate (MTX) in the treatment of active rheumatoid arthritis.

Studies abroad have shown that etanercept (trade name: Enbrel) has more rapid effect, better curative effect than MTX, and good safety in the treatment of active moderate and severe rheumatoid arthritis. Meanwhile, after 52 weeks of treatment, subjects treated with etanercept combined with MTX showed significant improvement in the Sharp score of joints compared those treated with the single use of MTX. Imaging showed that combined treatment could effectively prevent bone and joint destruction. However, there is still a lack of relevant research of rhTNFR:Fc (trade name: Yisaipu) as etanercept biosimilar in China. The main purpose of this study is to investigate the efficacy of RhTNFR:Fc combined with MTX in the treatment of active rheumatoid arthritis in Chinese population and the effect of long-term treatment on arthrography.

**2. Trial objective:**

Compare the clinical efficacy and the influence on the joint radiology progression between different treatment groups of RhTNFR:Fc combined with MTX and between these groups and the MTX monotherapy.

**3. Trial design:**

A randomized, controlled, open and multi-center clinical study, which is a Phase IV clinical study

**3. Study subjects:**

Rheumatoid arthritis subjects who meet the inclusion/exclusion criteria randomly divided into 3 groups (Group A, Group B and Group C) using centre-stratified block-permuted randomization with 30 patients in each group and 90 patients in total.

4.1 Inclusion criteria:

Only those subjects who meet all of the following conditions could be included in the trial.

1. The subjects diagnosed with rheumatoid arthritis according to the revised 1987 American College of Rheumatology (ACR) Criteria, and with a disease duration of ≥ 6 months.
2. During the screening period and the baseline period, the swollen joint count (SJC) of the subjects should be ≥ 6 (66 joints counted), while the tender joint count (TJC) should be ≥ 8 (68 joints counted); ESR ≥ 28mm/h or CRP ≥ 20 mg/l in laboratory examination; and ≥ 1 bone erosion lesions revealed in X-ray films of both hands/wrists.
3. Subjects aged between 18 and 60 (inclusive) years old;
4. There has been no use of any marketed or non-marketed biological agents for rheumatoid arthritis (focusing on: etanercept, infliximab, adalimumab, tituximab[CD20 McAb], tocilizumab[IL-6 receptor Ab], abatacept[CTLA4-Ig], anakinra[IL-1 antagonist]) before screening;
5. Any DMARDs drugs used other than MTX have been stopped for 4 weeks before the trial; Oral cortical hormones (prednisone ≤ 10 mg/d or its equivalent) and NSAIDs (up to the maximum recommended dose) are allowed, but the dose should be stable for at least 4 weeks before the baseline period;
6. Women of childbearing potential agreeing to take effective methods of contraception during the trial;
7. The urine pregnancy test of women of childbearing age should be negative during screening.
8. Subjects willing to sign the informed consent form (ICF) and comply with the requirements of the study protocol;

4.2 Exclusion criteria: Subjects complying with any of the following conditions should be excluded from the trial.

1. Major surgery (including joint surgery) performed within 8 weeks before screening, or major surgery planned within 6 months after enrollment.
2. Patients with rheumatic autoimmune diseases other than rheumatoid arthritis: including systemic lupus erythematosus (SLE), mixed connective tissue disease (MCTD), scleroderma and polymyositis. However, subjects with Sjogren syndrome are allowed to participate in this trial. If ANA is positive, the possibility of SLE should be excluded based on the clinical conditions and the negative result of the anti-DNA antibody test.
3. Rheumatoid arthritis with wrist X-ray being Stage IV.
4. Patients having suffered or suffering from inflammatory arthritis other than rheumatoid arthritis (such as gout, reactive arthritis, psoriatic arthritis, seronegative spondyloarthropathy, Lyme disease).
5. Patients who have received any investigational drug within 4 weeks before screening (or 5 half-life periods, whichever is the longer).
6. Patients who have undergone any cell elimination therapy, including the investigational drug (e.g. CAMPATH, anti-CD4, anti-CD5, anti-CD3 and anti-CDl9).
7. Patients treated with intravenous gamma globulin, plasma exchange or Prosorba plasma separation exchange within 6 months before the baseline,.
8. Patients who have been injected with cortical hormones intraarticularly or by other means within 4 weeks before the baseline.
9. Patients who have received any vaccine within 4 weeks before the baseline.
10. Patients who have used any alkylating agents, such as cyclophosphamide, chlorambucil or total lymphoid irradiation, within 4 weeks before the baseline.
11. Subjects with severe and uncontrolled cardiovascular diseases, neurological diseases, pulmonary diseases (including obstructive pulmonary disease and interstitial lung disease), nephropathy, liver diseases, endocrine diseases (including uncontrolled diabetes) and gastrointestinal diseases, etc.
12. Patients with uncontrolled disease state, such as asthma, psoriasis, inflammatory bowel disease and other diseases, usually requiring oral administration or injection of cortical hormones to treat recurrence.
13. Patients with known currently active or recurrent bacterial, viral, fungal, mycobacterial diseases or other infectious diseases (including but not limited to tuberculosis and atypical mycobacterial diseases, granulomatous diseases shown by chest X-ray, hepatitis B and C, HIV infection, herpes zoster, but excluding onychomycosis infection), or patients requiring hospitalization and intravenous antibiotic therapy within 4 weeks prior to screening or infected patients requiring oral antibiotic therapy within 2 weeks prior to screening.
14. Subjects with a history of malignant tumors, including solid tumors and hematological malignancies (except for patients with cutaneous basal cell carcinoma that has been excised or cured).
15. Pregnant or lactating (breastfeeding) women.
16. Subjects suffering from neuropathy and other painful diseases that may interfere with pain evaluation.
17. Serum creatinine > 1.5 mg/dl.
18. Alanine aminotransferase (ALT) or aspartate aminotransferase (AST) > 2 x ULN (if ALT or AST is measured to be > 2 x ULN for the first time, sampling should be conducted again during the screening period), or total bilirubin > 1 x ULN (if total bilirubin is measured to be > 1 x ULN for the first time, sampling should be conducted again during the screening period).
19. Platelet count < 100 × 10^9^ /L or WBC < 3 × 10^9^ /L.
20. HBsAg or anti-HCV positive.

**5. Test method and course of treatment**

5.1 Test method:

The trial is a randomized, controlled, open-label, multicenter clinical study. Qualified subjects after screening will be randomly divided into 3 groups (Groups A, B and C) using centre-stratified block-permuted randomization, with 30 patients in each group and 90 patients in total:

5.2 Drug usage:

1. In MTX therapy, the initial dose is 10mg/ week, which will gradually be increased to 15 mg/ week (in Week 4);
2. At the same time, 25mg of RhTNFR:Fc (trade name: Yisaipu) will be injected subcutaneously each time, twice a week for treatment.
3. The subjects will be treated with the combined use of MTX according to the above regimen until Week 52, and endpoint evaluation will be conducted. The dose of MTX could be reduced for safety reasons. If the subjects could not tolerate the dose reduction, the event could be recorded as an adverse event. After reduction, the dose could be restored to the prescribed dosage when the investigator thinks it is appropriate.
4. Diagram of drug usage:

Group A:

(RhTNFR:Fc + MTX group), 52 weeks; n=30

Group B:

"MTX monotherapy"; n=30

(RhTNFR:Fc + MTX group)

Group C:

"MTX monotherapy"; n=30

Baseline

52 weeks

24 weeks

4 weeks

12 weeks

36 weeks

MTX dose increase stage: MTX will be increased from 10 mg/ week to 15 mg/ week

Note: Folic acid at 5 mg/ week will be given on the Day 2 of MTX administration in the trial.

5.3 Criteria for drug withdrawal:

If one of the following conditions occurs, the investigator may consider drug discontinuation for observation:

1. A serious adverse event occurs, and it has certain relationship with the investigational drug;
2. An adverse drug reaction occurs, and effective remission cannot be achieved after symptomatic treatment;
3. Laboratory tests: transaminase > 3 times the normal value or WBC < 3×10^9^ /L, or PLT < 100×10^9^ /L;
4. The subject has other diseases, and the investigational drug may aggravate the condition.

5.4 Withdrawal from the trial:

For the patients who withdraw during the trial, the investigator must record the reason and date of withdrawal in detail. Patients who may withdraw during the trial can be divided into the following categories:

- 1. Patients found to fail to meet the inclusion/exclusion criteria during the trial;
  2. Treatment discontinuation > 4 weeks during the medication due to adverse drug reaction or other reasons (e.g. the treatment has to be discontinued due to occurrence of other disease, migration to other places and loss to follow-up); or within 24 weeks, the accumulated duration of drug discontinuation has exceeded 8 weeks;
  3. Subjects who withdraw the drug by themselves and give up continuing the trial;
  4. Due to serious adverse drug reaction, the drug must be withdrawn in advance.
  5. Those with poor compliance and failing to take the drug according to the schedule or design;
  6. Those with concurrent occurrence of other serious diseases and the investigator considers inappropriate to continue the medication;
  7. Those failing to complete all the examination items as required and thus not evaluable.

5.5 Trial assessments:

The screening visit will be completed within 3 weeks (21 days) before the first administration of the investigational drug. The baseline visit and randomization will be conducted on the day of the first administration, and informal visits will be conducted every 4 weeks to understand the medication and adverse events of the subjects. Formal visits will be conducted every 12 weeks, and the visit results will be recorded. The endpoint visits will be conducted at week 52. In addition to the baseline, the time window for each visit is ±7 days (based on the baseline).

5.6 Standardized sequence of visit evaluation

1. Health assessment of subjects (HAQ)
2. Measurement of subjects' own assessment (VAS for pain and VAS for overall assessment of disease activity)
3. Therapists or joint assessors:
   1. Counting of affected joints
   2. Overall evaluation of disease activity VAS and safety (adverse events, vital signs, concomitant medication, laboratory data review).
4. The therapists prescribe tests and special examinations, guiding the subjects to draw blood and receive X-ray and other laboratory examinations.

5.7 Assessment personnel

1. Joint assessors:

Rheumatologists or experienced arthritis assessors who have no right to know about other data information of the subjects. At each visit, each subject will be evaluated by the same joint evaluator.

1. Principal Investigator:

Rheumatologists who have the right to know about other data information of the subjects, and could make all treatment decisions according to the clinical treatment response and laboratory indexes of the subjects.

**6. Efficacy observation indicators:**

6.1 Clinical observation indicators and assessments:

6.1.1 Rest pain:

Using 100 mm visual analog scale, each subject indicates the degree of pain;

**
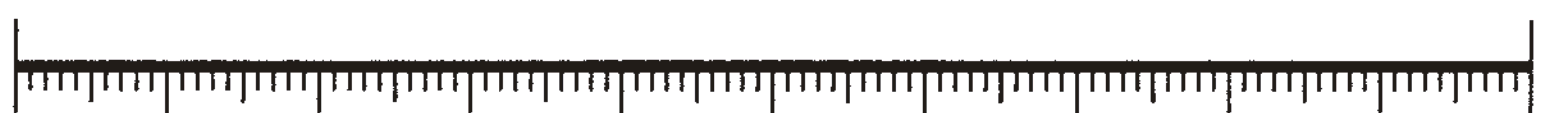
**

**10**

**0**

**20**

**30**

**40**

**50**

**60**

**70**

**80**

**90**

**100 mm**

6.1.2 Duration of morning stiffness (min):

Each subject records the time from the occurrence to the disappearance of joint stiffness when waking up in the morning;

6.1.3 Joint swelling:

The swollen joint count will be recorded (SJC, 66 joints counted)

6.1.4 Joint tenderness:

The tender joint count will be recorded (TJC, 68 joints counted)

6.1.5 Health Assessment Questionnaire (HAQ):

Before and after treatment, each subject assesses his/her own ability of daily living, including 20 indicators, such as dressing, standing, eating and drinking, walking, hygiene and grasping. The assessment includes 4 levels: 0= not difficult; 1= slightly difficult; 2=very difficult; 3= incapable. See the attachments for details.

6.1.6 Subject's assessment on the current disease status:

The 100 mm visual analog scale is used to assess the comprehensive disease condition before and after medication. The following way can be used for asking: “Considering all factors of arthritis that affect you, please indicate your current status in the scale with ‘X’”. The following degree classification can be used in combination for assessing the disease degree.

**
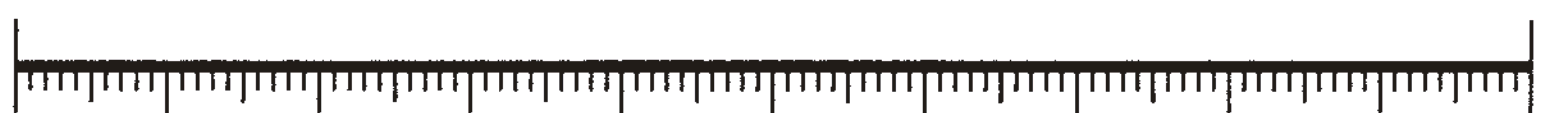
**

**10**

**0**

**20**

**30**

**40**

**50**

**60**

**70**

**80**

**90**

**100 mm**

6.1.7 Doctor's assessment on the current disease status:

The 100 mm visual analog scale is used to assess the comprehensive disease condition before and after medication. The method of assessment is the same as above.

6.2. Conventional laboratory indicators:

The blood routine examination, biochemical examination, erythrocyte sedimentation rate test (ESR, Wechsler method, normal values: ≤ 15 mm/h for males, ≤ 20 mm/h for females) and C-reactive protein test (CRP, quantitative method) will be conducted before treatment and at week 12, 24, 36 and 52 after treatment.

The urine routine examination will be conducted before treatment and at week 12, 24, 36 and 52 after treatment.

The rheumatoid factor (RF) examination and ANA will be conducted before treatment, and at 24 and 52 weeks after treatment.

The anti-CCP antibody test (2nd generation) will be conducted before treatment.

6.3. Safety observation indicators:

PPD, HbsAg antibody, HCV-Ab and HCV antibody were checked before enrollment. The laboratory indexes to be monitored in the trial included: blood routine, urine routine, liver and kidney function, ANA and chest radiography.

6.4. Imaging observation indicators:

The examinations for both hands include X-ray and MRI of wrist joints, and other parts will be examined if necessary. The Sharp score is used to evaluate the affected joints.

**7. Efficacy evaluation:**

7.1 Primary endpoints:

Improvement in Sharp score of X-ray of affected joints.

7.2 Secondary endpoints:

1. Proportion of subjects reaching ACR20, ACR50 and ACR70: ACR50 and ACR70 are defined as 50% and 70% improvement respectively using the same criteria as ACR20.
2. Average changes of ACR core parameters relative to the baseline level
3. Average change of HAQ score from baseline to Week 52
4. Time for each treatment group to reach ACR20, 50 and 70
5. The proportion of subjects with low disease activity at each visit
6. The proportion of subjects achieving disease remission at each visit
7. MRI changes (joint space narrowing and bone erosion)

7.2.1 The response definition of ACR20 is: there is 20% improvement in the number of swollen and tender joints (28), as well as 20 % improvement in at least 3 of the 5 following items:

1. The VAS score of pain assessed by the subject;
2. The VAS score of overall disease condition assessed by the subject;
3. The VAS score of overall disease condition assessed by investigator;
4. Health Assessment Questionnaire (HAQ);
5. Acute phase reactant (ESR or CRP).

7.2.2 DAS28:


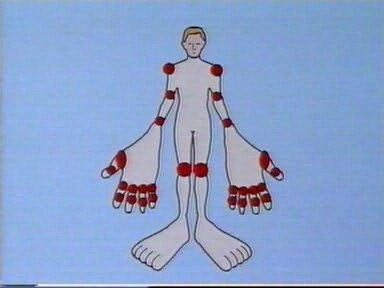
(28 joints)

The disease activity score of 28 joints will be calculated as follows:

DAS28 = 0.56 ×$\sqrt{\text{TJC}}$ + 0.28 × $\sqrt{\text{SJC}}$ + 0.7 × ln(ESR) + 0.014 × GH

TJC: Tender joint count, SJC: Swollen joint count, ESR: erythrocyte sedimentation rate (in mm/h), GH: general health status expressed in the 100 mm VAS.

**8 Safety evaluation:**

8.1 Nature and frequency of adverse reactions and events.

Adverse reaction assessment: The investigator assesses and records the severity of discomfort according to with following criteria, and the subjects should visit the doctor’s office regularly or at any time:

0= no discomfort;

1= slight discomfort, not affecting daily life and work;

2= moderate discomfort, affecting daily life and work;

3= severe discomfort, significantly affecting life and fitness status (requiring bed rest);

4= Life-threatening.

8.2 Tolerability assessment:

After the end of treatment, the physician in charge will conduct comprehensive evaluation on the tolerability of each subject according to the following criteria;

0= well tolerated, with no adverse reaction;

1= good tolerance, with slight adverse reaction which does not need treatment;

2= moderate tolerance, with adverse reaction needing to be treated;

3= poor tolerance, with adverse reaction which results in drug discontinuation.

8.3 Unexpected events:

The unexpected events occurring to the subjects during the course of treatment, such as cerebrovascular accident, automobile accident and death should be recorded.

8.4 Adverse event relevance:

The investigator should evaluate the relationships between adverse events (AEs) and the investigational drug / concomitant medication by reference to the 5-grade classification criteria below:

1) Definitely related: the reaction occurs following a reasonable temporal sequence from administration of the drug and falls into the known reaction types of the suspected drug; the reaction improves after drug withdrawal but occurred again after resumption of drug administration.

2) Possibly related: the reaction occurs following a reasonable temporal sequence from administration of the drug and falls into the known reaction types of the suspected drug; the reaction could also be caused by the subject’s clinical state or other therapeutic methods.

3) Possibly unrelated: the reaction occurs not quite following a reasonable temporal sequence from administration of the drug and does not fall into the known reaction types of the suspected drug completely; the reaction could also be caused by the subject’s clinical state or other therapeutic methods.

4) Definitely unrelated: the reaction occurs not following a reasonable temporal sequence from administration of the drug and falls into the known reaction types of other drugs than the investigational drug; the reaction could also be caused by the subject’s clinical state or other therapeutic methods, and the disease condition improves or symptoms of the reaction disappears after withdrawal of other therapeutic methods but appears again after resumption of other therapeutic methods.

5) Not evaluable: the reaction occurs not following a definite temporal sequence from administration of the drug and resembles the known reaction types of the drug, but it could also be caused by other drugs used at the same time.

The 1), 2) and 5) of the above 5 items are recorded as adverse reactions (ARs) of the drug.

Incidence of ARs: Number of patients experiencing ARs / Total number of patients × 100%.

8.5 Assessment of infection and other serious adverse events (SAEs):

Close attention should be paid to the occurrence of infection during observation and make statistics accordingly; in case of any serious adverse reaction (SAR) (e.g., serious infection and serious local reaction), the drug should be withdrawn immediately, the sponsor contacted as soon as possible and symptomatic treatment given promptly. If it is a mild or moderate AR, symptomatic treatment may be given first for observation; if the symptom is still not alleviated, the drug should be withdrawn and the changes in the disease condition should continue to be observed.

Re-examination should be performed immediately if the laboratory findings are slightly abnormal, e.g., elevated transaminase level; if the abnormalities persist, symptomatic treatment should be given for observation, and observation of changes in the disease conditions continued. The drug should be withdrawn immediately if the abnormalities continue to worsen or serious abnormalities occur.

8.6 Assessment of SAEs:

SAE is defined as any AE that occurs at any dose of the investigational drug or at any time during observation, including those that are immediately life-threatening, necessitate hospitalization or prolonged hospitalization, lead to disability, or cause congenital malformation or permanent loss of function.

In case of serious adverse events (related or unrelated to drugs), they should be reported to the Medical department of Shanghai CP Guojian Pharmaceutical Co., Ltd. and to the China Food and Drug Administration within 24 hours, and a written report should be made within 7 days.

**9. Statistical methods:**

9.1 Selection of statistical analysis data:

Intent-to-treat (ITT): all subjects eligible and enrolled after screening and having taken at least one dose of the drug and received at least one post-treatment effectiveness evaluation; LOCF (last observation carry-forward) will be adopted for those subject’s data not observed during the whole treatment course, that is, estimating the missing values of important indicators using the closest observed data. The ITT analysis will be used in this trial.

Safety population: it includes all subjects having taken at least one dose of the drug and received at least one post-treatment safety evaluation.

9.2 Statistics Analysis Plan:

Statistical analysis will be carried out using the SAS statistical analysis software. A sample size of 90 patients was estimated for the open label period with the assumption that mean mTSS CRF was -0.5±1.0 in the MTX plus rhTNFR:Fc group, and 2.5±5.0 in the MTX group at week 52. A sample size of 25 patients per treatment group was calculated to be necessary for more than 80% power to reject the null hypothesis of no difference among the treatment groups with an α of 0•05. Assuming loss of patients, the study required a minimum of 30 patients in each group. Two-sided test will be used for all statistical tests, and the difference tested will be considered as statistically significant if the P value is equal to or less than 0.05. Measurement data will be statistically described using mean±SD (standard deviation). Paired t-test will be used for intra-group differences before and after the treatment. Enumeration data will be statistically described using frequency (constituent ratio).

Dropout analysis: Calculate the dropout rates and the rates of dropout due to AE.

Efficacy analysis: The paired t test or nonparametric test or chi-square test will be used to evaluate the efficacy indexes.

Safety analysis: Calculate the incidence rate of adverse events and tabulate the AEs occurring during the trial, normal/abnormal changes in laboratory findings before and after the trial, as well as relationships of abnormal changes with the investigational drug

**10. Distribution of patients at various sites and duration of study:**

10.1 Distribution of patients:

| **Hospital number** | **Hospital** | **Person in charge** | **Number of cases** |
| --- | --- | --- | --- |
| 01 | Peking Union Medical College Hospital | Zhao Yan | **Competitive enrollment** |
| 02 | Peking University First Hospital | Zhang Zhuoli |
| 03 | Bethune International Peace Hospital | Li Zhenbin |
| 04 | Nanjing Drum Tower Hospital | Sun Lingyun |
| 05 | The Second Affiliated Hospital of Sun Yat-sen University | Dai Lie |
| 06 | West China Hospital of Sichuan University | Liu Yi |
| 07 | China-Japan Union Hospital of Jilin University | Bi Liqi |
|  | **Total** |  | **90** |

10.2 Study progress:

Initiation of study: April 2010

Subject inclusion: April 2010

End of subject inclusion: October 2010

End of observation: October 2011

Statistical analysis: November 2011

Summary report: December 2011

Attachment I: Trial Flow Chart

Attachment II: Health Assessment Questionnaire (HAQ)

Attachment III: Sharp Score Criteria

Attachment IV: Wrist X-ray Staging Criteria for Rheumatoid Arthritis (ACR)

Attachment V: Table of Swollen and Tender Joint Count

**Attachment I:**

**Trial Flow Chart**

| **Work Contents** | **Baseline Period** | **Treatment Period** | | | |
| --- | --- | --- | --- | --- | --- |
|  | **D-7~0** | **12W±7d** | **24W±7d** | **36W±7d** | **52W±7d** |
| **Informed consent** | **√** |  |  |  |  |
| **Inclusion criteria** | **√** |  |  |  |  |
| **Exclusion criteria** | **√** |  |  |  |  |
| **Medical history collection** | **√** |  |  |  |  |
| **Vital signs** | **√** | **√** | **√** | **√** | **√** |
| **Physical examination** | **√** | **√** | **√** | **√** | **√** |
| **HBs-Ag/HCV-Ab** | **√** |  |  |  |  |
| **Tuberculin test (PPD)** | **√** |  |  |  |  |
| **Chest X ray** | **√** |  |  |  | **√** |
| **Disease status assessment** | **√** | **√** | **√** | **√** | **√** |
| **Routine blood test** | **√** | **√** | **√** | **√** | **√** |
| **Urinalysis** | **√** | **√** | **√** | **√** | **√** |
| **Blood chemistry test** | **√** | **√** | **√** | **√** | **√** |
| **Erythrocyte sedimentation rate (ESR)** | **√** | **√** | **√** | **√** | **√** |
| **C-reactive protein (CRP)** | **√** | **√** | **√** | **√** | **√** |
| **Rheumatoid factor** | **√** |  | **√** |  | **√** |
| **Anti-CCP (2nd Generation) antibody** | **√** |  |  |  |  |
| **Antinuclear antibody (ANA)** | **√** |  | **√** |  | **√** |
| **ECG** | **√** | **√** | **√** | **√** | **√** |
| **X-ray of both hands (including wrist joint)** | **√** |  | **√** |  | **√** |
| **MRI** | **√** |  |  |  | **√** |
| **Medication administration record** |  | **√** | **√** | **√** | **√** |
| **Documentation of concomitant medications** | **√** | **√** | **√** | **√** | **√** |
| **Adverse event evaluation** |  | **√** | **√** | **√** | **√** |
| **Clinical efficacy evaluation** |  | **√** | **√** | **√** | **√** |

**Attachment II:**

**Health Assessment Questionnaire (HAQ)**

Each subject assesses his/her own ability of daily living, including 20 indicators such as dressing, standing, eating and drinking, walking, hygiene, grasping. The assessment is divided into four levels:

0= not difficult; 1= slightly difficult; 2=very difficult; 3= incapable.

| **When doing the following activities in the past week:** | | **Score** |
| --- | --- | --- |
| 01 | Put on clothes (Fasten shoelaces and buttons)? |  |
| 02 | Comb your hair? |  |
| 03 | Stand up from a chair without support from hands? |  |
| 04 | Get into bed, get out of bed? |  |
| 05 | Raise a glass to drink water? |  |
| 06 | Cut vegetables? |  |
| 07 | Unscrew a bottle cap? |  |
| 08 | Walk on the flat ground outdoors? |  |
| 09 | Ascend the stairs for 5 steps? |  |
| 10 | Dry yourself after bathing? |  |
| 11 | Get up from the toilet seat or sit down by yourself? |  |
| 12 | Bend down to pick up things on the floor? |  |
| 13 | Reach out for the clothing on the coat rack? |  |
| 14 | Turn on and off a water faucet? |  |
| 15 | Get on and off (buses, cars)? |  |
| 16 | Shop around? |  |
| 17 | Do household chores like cleaning the floor? |  |
| 18 | Walk for 1 kilometer? |  |
| 19 | Participate in an activity you enjoy? |  |
| 20 | Sleep well at night? |  |
| **Total score** | |  |
| **Average score** | |  |

**Attachment III:**

**Sharp Score Criteria**

**Joint Erosion Score (ES)**

The simple scoring method is: The joint is divided into 4 equal parts, and 1 point is given if there is erosion in each part. If there is an intersection part, it is scored according to one fifth of the eroded area of the joint, with one point for every one fifth.


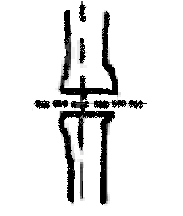


With the erosion scores being 0-5, the judgment criteria are as follows:

0 = no erosion;

1 = 1 quarter joint part is eroded alone, or the eroded area of the joint is ≤ 20%;

2 = 2 quarter joint parts are eroded respectively, or 20% < joint eroded area ≤ 40%;

3＝3 quarter joint parts are eroded respectively, or 40% < joint eroded area ≤ 60%；

4＝4 quarter joint parts are eroded respectively, or 60% < joint eroded area ≤ 80%；

5 = the joint is completely destroyed.

**Joint Space Narrowing Score, JSN:**

The JSN scores are 0-4.

0= normal

1 = punctiform protrusion, asymmetric, with the narrowing degree < 25%;

2 = Narrowing degree of 25-60%;

3＝Narrowing degree of 60-99％；

4 = complete rigidity or dislocation.

**Attachment IV:**

**Wrist X-ray Staging Criteria for Rheumatoid Arthritis (ACR)**

| Level I: Osteoporosis of joints or under articular surfaces. |
| --- |
| Level II: Subarticular osteoporosis, with occasional cystic destruction of articular surface or bone erosion and destruction. |
| Level III: Obvious articular surface damage or bone erosion damage, joint space narrowing, joint subluxation and other changes. |
| Level IV: Other lesions than Phase II and III lesions, with fibrous or bony rigidity. |

**Attachment V: Joint swelling and tenderness**

The swollen and tender joint count will be recorded (SJC, 66 joints counted; TJC, 8 joints counted):

Table of Swollen and Tender Joint Count

| Right side | | | Joint | Left side | | |
| --- | --- | --- | --- | --- | --- | --- |
| Tender  1 2 3 | Swelling  1 2 3 | NE |  | NE | Tender  1 2 3 | Swelling  1 2 3 |
| □ □ □ | □ □ □ | □ | Temporal-mandibular joint | □ | □ □ □ | □ □ □ |
| □ □ □ | □ □ □ | □ | Sternoclavicular joint | □ | □ □ □ | □ □ □ |
| □ □ □ | □ □ □ | □ | Acromioclavicular joint | □ | □ □ □ | □ □ □ |
| □ □ □ | □ □ □ | □ | ***Shoulder joint*** | □ | □ □ □ | □ □ □ |
| □ □ □ | □ □ □ | □ | ***Elbow joint*** | □ | □ □ □ | □ □ □ |
| □ □ □ | □ □ □ | □ | ***Wrist joint*** | □ | □ □ □ | □ □ □ |
| □ □ □ | □ □ □ | □ | ***First metacarpophalangeal joint*** | □ | □ □ □ | □ □ □ |
| □ □ □ | □ □ □ | □ | ***Second metacarpophalangeal joint*** | □ | □ □ □ | □ □ □ |
| □ □ □ | □ □ □ | □ | ***Third metacarpophalangeal joint*** | □ | □ □ □ | □ □ □ |
| □ □ □ | □ □ □ | □ | ***Fourth metacarpophalangeal joint*** | □ | □ □ □ | □ □ □ |
| □ □ □ | □ □ □ | □ | ***Fifth metacarpophalangeal joint*** | □ | □ □ □ | □ □ □ |
| □ □ □ | □ □ □ | □ | ***Thumb interphalangeal joint*** | □ | □ □ □ | □ □ □ |
| □ □ □ | □ □ □ | □ | ***Second proximal interphalangeal joint*** | □ | □ □ □ | □ □ □ |
| □ □ □ | □ □ □ | □ | ***Third proximal interphalangeal joint*** | □ | □ □ □ | □ □ □ |
| □ □ □ | □ □ □ | □ | ***Fourth proximal interphalangeal joint*** | □ | □ □ □ | □ □ □ |
| □ □ □ | □ □ □ | □ | ***Fifth proximal interphalangeal joint*** | □ | □ □ □ | □ □ □ |
| □ □ □ | □ □ □ | □ | Second distal interphalangeal joint | □ | □ □ □ | □ □ □ |
| □ □ □ | □ □ □ | □ | Third distal interphalangeal joint | □ | □ □ □ | □ □ □ |
| □ □ □ | □ □ □ | □ | Fourth distal interphalangeal joint | □ | □ □ □ | □ □ □ |
| □ □ □ | □ □ □ | □ | Fifth distal interphalangeal joint | □ | □ □ □ | □ □ □ |
| □ □ □ | □ □ □ | □ | Hip joint | □ | □ □ □ | □ □ □ |
| □ □ □ | □ □ □ | □ | ***Knee joint*** | □ | □ □ □ | □ □ □ |
| □ □ □ | □ □ □ | □ | Ankle joint | □ | □ □ □ | □ □ □ |
| □ □ □ | □ □ □ | □ | Tarsal joint | □ | □ □ □ | □ □ □ |
| □ □ □ | □ □ □ | □ | First metatarsophalangeal joint | □ | □ □ □ | □ □ □ |
| □ □ □ | □ □ □ | □ | Second metatarsophalangeal joint | □ | □ □ □ | □ □ □ |
| □ □ □ | □ □ □ | □ | Third Metatarsophalangeal joint | □ | □ □ □ | □ □ □ |
| □ □ □ | □ □ □ | □ | Fourth metatarsophalangeal joint | □ | □ □ □ | □ □ □ |
| □ □ □ | □ □ □ | □ | Fifth metatarsophalangeal joint | □ | □ □ □ | □ □ □ |
| □ □ □ | □ □ □ | □ | First interphalangeal joint | □ | □ □ □ | □ □ □ |
| □ □ □ | □ □ □ | □ | Second interphalangeal joint | □ | □ □ □ | □ □ □ |
| □ □ □ | □ □ □ | □ | Third interphalangeal joint | □ | □ □ □ | □ □ □ |
| □ □ □ | □ □ □ | □ | Fourth interphalangeal joint | □ | □ □ □ | □ □ □ |
| □ □ □ | □ □ □ | □ | Fifth interphalangeal joint | □ | □ □ □ | □ □ □ |

**Note: ①1= Yes, 2= No, 3= Not done; ② NE = Not Evaluable;**
